# Evidences for Expression and Location of ANGPTL8 in Human Adipose Tissue

**DOI:** 10.3390/jcm9020512

**Published:** 2020-02-13

**Authors:** Leonardo Catalano-Iniesta, Virginia Sánchez Robledo, María Carmen Iglesias-Osma, Amparo Galán Albiñana, Sixto Carrero, Enrique J. Blanco, Marta Carretero-Hernández, José Carretero, María José García-Barrado

**Affiliations:** 1 Department of Physiology and Pharmacology, Faculty of Medicine, University of Salamanca, 38007 Salamanca, Spain; leonardo.catalano@usal.es (L.C.-I.); robledo@usal.es (V.S.R.); mcio@usal.es (M.C.I.-O.); 2 Laboratory of Neuroendocrinology, Institute of Neurosciences of Castilla y León (INCyL), and Laboratory of Neuroendocrinology and Obesity, Institute of Biomedical Research of Salamanca (IBSAL), University of Salamanca, 38007 Salamanca, Spain; ejbb@usal.es (E.J.B.);; 3 Gene Expression and RNA Metabolism Laboratory, Centro de Investigación Príncipe Felipe (CIPF), C/ E. Primo Yúfera 3, 46012 Valencia, Spain; agalan@cipf.es; 4 Surgery Service of University Hospital of Salamanca, 38007 Salamanca, Spain; scarrero55@gmail.com; 5 Department of Human Anatomy and Histology, Faculty of Medicine, University of Salamanca, 38007 Salamanca, Spain; martataes@gmail.com

**Keywords:** ANGPTL8, visceral adipose tissue (VAT), obesity, endothelial cells

## Abstract

The metabolism of triglycerides (TGs) is regulated, among others, by the lipoprotein lipase (LPL) that hydrolyses the TGs on endothelial cells. In turn, LPL is inhibited by the ANGPTLs family of proteins, such as ANGPTL3, 4, and, 8; the latter is the least known. In this work, we have tried to establish the expression and localisation of the Angiopoietin-like 8 (ANGPTL8) protein in the visceral adipose tissue (VAT) of morbid-obese and non-obese patients. 109 subjects (66 women and 43 men) undergoing laparoscopic surgery participated in this study. A blood sample and a portion of the VAT were obtained, and the patients were classified according to their Body Mass Index (BMI) as non-obese (19.5–30 kg/m^2^) and morbid-obese (40–50 kg/m^2^). No significant changes in ANGPTL8 plasma levels were determined by EIA in obese patients. The immunocytochemistry and Western blotting showed the presence of increased ANGPTL8 in morbid-obese patients (*p* < 0.05). In-situ hybridisation and a real time polymerase chain reaction (RT-PCR) confirmed that the mRNA that encodes ANGPTL8 was present in adipocytes, without differences in their nutritional state (*p* = 0.89), and even in the endothelial cells. Our data suggests that ANGPT8 plasmatic levels do not change significantly in patients with morbid obesity, although there is a modest difference related to gender. Besides, we demonstrate that in visceral adipose tissue, ANGPTL8 is well defined in the cytoplasm of adipocytes coexisting with perilipin-1 and its mRNA, also is present in endothelial cells. These findings suggest the possibility that among other functions, ANGPTL8 could perform either a paracrine and/or an endocrine role in the adipose tissue.

## 1. Introduction

The prevalence of obesity has grown in an epidemic way, and nowadays millions of people suffer from this disease. In addition, excess weight is associated with other pathologies, such as type 2 diabetes, cardiovascular disease, metabolic syndrome and some types of cancer [1]. It is widely recognised by both epidemiological and controlled clinical trials, that obesity is often associated with hypertriglyceridaemia with lower levels of HDL-cholesterol and, sometimes, higher LDL-cholesterol. Hypertriglyceridaemia is a common element in the pathologies closely related to obesity [2]. 

Patients with genetic mutations of lipoprotein lipase (LPL) present severe hypertriglyceridaemia that evidenced the relevance of LPL [3]. Similarly, the disruption of the COOH–terminal in the LPL, in mice, causes remarkably higher plasma triglycerides (TGs) levels at birth, and the deaths of animals occur within the first 24 h [4]. Conversely, transgenic mice that overexpress human LPL throughout the body show a 75% reduction in plasmatic TG [5]. Therefore, LPL activity is carefully regulated to ensure that the rate of the uptake of fatty acids matches local energy demands.

A significant group of physiological regulators of LPL activity are the angiopoietin-like proteins, including the ubiquitously expressed angiopoietin-like 3, 4 and 8 [6]. It is known that the ANGPTL protein family, mainly angiopoietin-like 3 and 4, participates in lipid metabolism, since it has been shown to inhibit lipoprotein lipase [7,8]. The ANGPTL8, the last member of the group of angiopoietin-like proteins, was discovered and reported by several groups in 2012, with different denominations, such as RIFL [9], lipasin [10], Angptl8 [11] and betatrophin [12]. ANGPTL8 presents differences with other members of this group because it lacks the characteristic C-terminal fibrinogen-like domain, although it is speculated that it has similar properties to the rest of the angiopoietin-like proteins [13,14].

An important role in the proliferation of pancreatic beta cells was attributed to ANGPTL8 [12]. However, after controversial experimental data, this effect has been discarded [15], and there is unanimous agreement that it does not control the proliferation of beta cells in the mouse model [16,17]. Besides, the findings obtained that correlate ANGPTL8 plasma levels and diabetes control are also discrepant [18,19,20,21,22,23,24,25]. The association of ANGPTL8 with insulin resistance [18,26,27], blood glucose [28,29], C-peptide and glycosylated haemoglobin (HbA1C) have not offered clear results [30]. In animals, a positive correlation of circulating ANGPTL8 with triglyceride levels exists [10,31,32,33]. These facts were also confirmed in humans [34].

The role of ANGPTL8 in the regulation of lipid metabolism is the main object of many studies; furthermore, there are several theories regarding its possible mechanism of action [35,36]. One model proposes that ANGPTL3, 4 and 8 proteins negatively regulate LPL activation depending on the nutritional status and the tissue on which they act. In cardiac and skeletal muscle, ANGPTL3 requires ANGPT8 to inhibit LPL activity during the feeding cycle to direct circulating TG to white adipose tissue (WAT) for storage. During fasting there is induced ANGPTL4 and suppressed ANGPL8, which implies that the TGs are directed to the muscles, though this model does not explain the functional role of ANGPTL8 in adipose tissue [35].

In this work, we have tried to establish the expression and localisation of the ANGPTL8 protein in visceral adipose tissue of morbid-obese and non-obese patients, to find out what function it could play in lipid metabolism.

## 2. Experimental Section

### 2.1. Study Population and Ethical Statement

The present study was approved by the Institutional Research Ethics Committee of the University Hospital of Salamanca, and follows the ethical guideline of the declaration of Helsinki. All participants were supplied with oral and written information about the study, and they gave written consent. 109 subjects (66 women and 43 men) undergoing laparoscopic surgery were recruited, all of them classified according to their Body Mass Index (BMI) in being non-obese (19.5–30 kg/m^2^) and morbid-obese (40–50 kg/m^2^). The inclusion criteria were (1) age 18–65 years; (2) Non-diagnosis and treatment of diabetes; (3) no known acute or chronic disease except for obesity based on history, physical examination and standard laboratory tests; (4) the patients included within the obesity group were undergoing bariatric surgery. Consequently, 42 non-obese and 67 morbid-obese patients were included in this study.

### 2.2. Anthropometric and Biochemical Measurements. Sample collection

Anthropometric measurements such as height, weight and waist circumference were recorded in all participants wearing light indoor clothing and barefooted. Height was measured to the nearest 0.1 cm with a Holtain stadiometer (Holtain Ltd, Crosswell, UK), whereas body weight was measured with a calibrated electronic scale to the nearest 0.1 kg. Waist circumference was measured at the midpoint between the iliac crest and the rib cage on the midaxillary line. 

Before laparoscopy surgery, fasting blood samples were taken using vacutainer^®^ EDTA tubes. Plasma was obtained after centrifugation for 10 min at 3000× *g* at 4 °C, and then aliquoted and stored at −80 °C until assayed. Glucose and lipid profiles (glycaemia, insulinaemia, HbA1c, total TG, low and high-density lipoprotein cholesterol concentrations, and leptin levels) were analysed in the Biochemical Laboratory of the University Hospital of Salamanca using routine procedures. Parameters derived from the HOMA2 index were calculated with the HOMA2 calculator system of the University of Oxford (Diabetes Trials Unit 2004).

At the time of the laparoscopy surgery, a portion of the visceral adipose tissue (VAT) was obtained. The samples of VAT were washed with phosphate buffered saline (PBS) before their processing. Half of the adipose tissue specimen was fixed in formaldehyde 4% in PBS (0.1 M, pH 7.4, 0.8% NaCl) for 24 h at 4 °C, and then washed in PBS for 24 h for histological study. The rest was immediately frozen in liquid N_2_ for Western blotting and the real time polymerase chain reaction (RT-PCR).

### 2.3. Enzyme Immunoassay of ANGPTL8 in Human Plasma

The plasma samples were thawed on ice and centrifuged at 10,000× *g* for 5 min at 4 °C to remove any debris. C-terminal 139–198 form of human ANGPTL8 (betatrophin) levels in circulation was measured using an enzyme immunoassay kit (EIA) recognising the ANGPTL8 region spanning from 139 to 198 amino acid (Phoenix Pharmaceuticals®, Karlsruhe, Germany, catalogue number EK-051-55). No significant cross reactivity with other proteins was observed. Intra-assay variation was < 10%, and inter-assay coefficients of variation were <15%. 

### 2.4. Immunohistochemistry

The fixed adipose tissue samples were dehydrated in ethanol and embedded in paraffin to obtain 5 µm serial sections for immunohistochemistry. The slides were previously deparaffinised and rehydrated. The endogenous peroxidase was blocked by incubating with 0.2% H_2_O_2_ in methanol for 30 min. Afterwards, they were washed three times in TBS (Trizma-HCl buffered saline 0.05 M, pH 7.4, plus 0.9% NaCl, used as the solution for washes and dilutions). The nonspecific reaction of the secondary antibody was blocked by incubation in normal swine serum (Dako^®^, Santa Clara, CA, USA, diluted 1:30 in TBS) for 30 min. In the first group of slides, a single immunostaining method was performed to detect ANGPTL8 in adipose tissue; the sections were incubated in the primary antibody, polyclonal rabbit anti-ANGPTL8 (Novus Biologicals^®^, Abingdon, UK, diluted 200 in TBS) for 24 h at 4 °C in a humidity chamber. After washing, the slides were incubated for 120 min at room temperature with goat anti-rabbit IgG Cy3 (1:800 in TBS, Abcam^®^, Cambridge, UK) After 5 min, Mayer’s haematoxylin was used to visualise the nuclei. Finally, the porta-objects were mounted with fluoromount aqueous mounting medium (Sigma^®^, St Louis, MO, USA). The fluorescence visualisation in slides was obtained by a confocal microscope TCSSP2 (Leica Biosystems^®^, Wetzlar, Germany). In the second group of slides, an immunofluorescence study was carried out to determine ANGPTL8, perilipin-1 and CD34. For this, sections were incubated with polyclonal rabbit anti-ANGPTL8 overnight at 4 °C in a humidity chamber, followed by incubation with goat anti-rabbit IgG Cy3 (1:800 in TBS, Abcam^®^) for 45 min at room temperature. Other sections were incubated with polyclonal rabbit anti-ANGPTL8, polyclonal goat anti-perilipin-1 (Abcam diluted 1:100 in TBS) and monoclonal antibody CD34 (QBEnd/10, LEICA Biosystems) overnight at 4 °C in a humidity chamber, followed by incubation with goat anti-rabbit IgG Cy3 (1:800 in TBS, Abcam^®^) and donkey anti-goat IgG Alexa fluor 488 (1:400 Abcam) for 120 min at room temperature. Slides were mounted with fluoromount aqueous mounting medium (Sigma^®^). The fluorescence visualisation in slides was obtained by a confocal microscope TCSSP2 (Leica^®^). 

### 2.5. In-Situ Hybridisation 

For determination of the expression of ANGPTL8 mRNA in human adipose tissue, in-situ hybridisation was performed by the immunocytochemical and immunofluorescence detection of biotin, using the Cy3-conjugated streptavidin method. For this study, the sense biotinylated oligonucleotide *GCC TGA ATC TGC CTG GAT GGA ACT GAG*, and antisense *CTC AGT TCC ATC CAG GCA GAT TCA GGC*, were used as probes 100% specific to human ANGPTL8. The screening was performed using the BLAST^®^ online application [37] to prevent the probe from joining any other chain with the same sequence that is not under study. 

The sections were previously deparaffinised and rehydrated, and then, were post-fixed in an acetylated buffer (75 mM triethanolamine, 25 mM acetic anhydride, 70 μM DEPC, 0.9 NaCl%, in distilled water, pH 7.5) for 10 min at room temperature to avoid the appearance of false positives. After several washes with phosphate buffered saline (1 mM KH_2_PO_4_, 5.5 mM Na_2_HPO_4_, 70 μM DEPC, 0.9% NaCl, in distilled water, pH 7.5) to remove traces of acetylated buffer, the samples were dried at 37 °C for 1 hour.

Afterwards, the slides were preincubated with a hybridisation buffer (Omnibuffer^®^, WAK-Chemie Medical GmbH, Steinbach, Germany) for 1 h at 39 °C. Hybridisation with the biotinylated-probe (25 ng/µL in Omnibuffer^®^) was carried out by using a Hybaid OmniSlide Thermal Cycler System (Fisher Scientific^®^, Waltman, MA, USA) overnight at 39 °C. The reaction was stopped by washing sequentially in high stringency conditions in standard saline citrate (SSC) (NaCl 0.6 M; Na_3_C_6_H_5_O_7_ 0,07 M; DEPC 1.5 mM, in distilled water pH 7), 4× SSC at 30 °C for 10 min, and afterward in 2× SSC, 1× SSC and 0.5× SSC at room temperature for 5 min. 

The next step was washing the samples with TrisEDTA saline buffer with ribonucleases (0.5 M NaCl, 15.5 mM Trizma base, 1 mM EDTA, 2% RNase A (Roche^®^, Barcelona, Spain) in distilled water, pH 8) for 30 min at 37 °C, and equilibrated for 5 min in Tris buffered saline (100 mM Tris–HCl, 150 mM NaCl, pH 7.5). Endogenous tissue immunoglobulins were blocked with normal swine serum (1:30 in TBS) for 30 min at room temperature. 

In the slides, biotin was detected using monoclonal anti-biotin antibody (DAKO^®^, Santa Clara, CA, USA, diluted 1:350 in TBS: 0.05 M HCl-Trizma, pH 7.4, plus 0.8% NaCl) overnight at 4 °C in a humidity chamber, and immunofluorescence was detected by Cy3-conjugated Streptavidin (Jackson ImmunoResearch^®^ Suffolk, UK, 3 µg/mL). Slides were mounted with fluoromount aqueous mounting medium (Sigma^®^). The fluorescence visualisation in slides was obtained by a confocal microscope TCSSP2 (Leica^®^). The slides were counterstained using Mayer’s acid haematoxylin. After 2 min, Mayer’s haematoxylin was used to visualise the nuclei. As controls, hybridisation with sense probe, omission of the probe and pre-treatment with RNase were performed, but a nonreaction was observed in any case. 

### 2.6. Western Blotting

Abdominal fat depots were collected and frozen immediately in liquid nitrogen as described above. Tissue was lysed in RIPA buffer (Nonidet P-40 1%; sodium deoxycholate 0.5%; SDS 0.1%; cocktail of protease inhibitors P8340 Sigma^®^, 1% in PBS pH 7.4) by polytron, and the homogenates were clarified by centrifugation at 16,000× *g* for 30 min at 4 °C. Protein determination was performed by the Lowry method [38]. 50 µg of total protein was separated by SDS-PAGE. Gels were transferred to Immun-Blot^TM^ PVDF Membrane (Bio-Rad Laboratories^®^, Barcelona Spain) and were incubated with the following antibodies: polyclonal rabbit anti-ANGPTL8 (Novus Biologicals^®^, Oxfordshire, UK) polyclonal rabbit ERK1/2 (Cell Signaling Technology^®^, Danvers, MA, USA). Secondary antibody was anti-rabbit HRP (Jackson ImmunoResearch^®^). Westerns were developed by ECL (Blotting Reagents, Sigma^®^). The images obtained were processed with the Multi Gauge program (Fujifilm^®^, Royston, UK, v 3.0). The control methods included the lack of the primary antibodies, pre-absorption tests with Betatrophin recombinant protein Human (ANGPTL8; Phoenix Pharmaceuticals^®^), as well as omission of the secondary antibody. After these tests, no immunoreactivity was detected. As a positive control of ANGPTL8 we used rat liver [9]. 

### 2.7. Gene Expression Analysis

Total RNA was extracted from abdominal fat using a combined protocol including Trizol (Sigma) and RNeasy Mini Kit (Qiagen^®^, Madrid, Spain) with DNaseI Digestion. First-strand synthesis was performed using EcoDry Premix (Takara BIO-Europe^®^, Saint-Germain-en-Laye, France) and RT-PCR was carried out in LightCycler 480 Instrument II^®^ (Roche, Barcelona, Spain) using SYBR^®^ PreMix ExTaqTM (Mi RNaseH Plus, Takara). ANGPTL8 gene expression analysis was performed using intron spanning primers (Forward: *5’- GGC AAG CCT GTT GGA GAC T*; Reverse: *5’- TGT CCC GTA GCA CCT TCT GT*), and relative gene expression was calculated by normalisation to Ribosomal Protein L19 (*RPL19*) (Forward: *5’- CGA ATG CCA GAG AAG GTC AC* and Reverse: *5’- CCA TGA GAA TCC GCT TGT TT*) as housekeeping in human adipose tissue.

### 2.8. Statistical Analysis

Statistical analyses were performed with GraphPad Prism 5 (GraphPad Software V 5.01). The results are expressed as arithmetic means ± the standard error of the mean (SEM). When two data sets were compared, Student’s *t*-test was used. Differences among groups were analysed using the one-way analysis of variance (ANOVA). In other cases, statistical significance among groups was analysed by two-way ANOVA, with gender and obesity as main factors. 

Spearman’s correlation analyses were carried out to determine the relationships between plasma concentrations of ANGPTL8, and the clinical parameters. The differences observed were considered significant when: *p* < 0.05 (*), *p* < 0.01 (**) and *p* < 0.001 (***).

## 3. Results

### 3.1. Clinical and Biochemical Characteristics of Non-Obese and Obese Subjects

Table 1 shows the anthropometric characteristics of the subject groups studied, separated by gender (women and men) and BMI (non-obese and morbid-obese). Our population was made up of 109 subjects, 66 of whom were women, 44 obese and 22 non-obese, and 43 were men, 23 obese and 20 non-obese. The average age of participants was 58 ± 14 and 48 ± 2 years old for non-obese and obese women, and 58 ± 8 and 47 ± 3 for non-obese and obese men, respectively. As expected, obese patients exhibited significant differences in BMI and waist circumference compared with non-obese patients (*p* < 0.001).

The clinical and biochemical parameters of the studied patients are shown in Table 2. The basal glycaemia was at normal levels in all of the groups studied. However, insulin levels and HbA1C were higher in obese patients, with a significantly increased insulinaemia, reduction of insulin sensitivity and increase of pancreatic beta cell function calculated by the homeostatic model HOMA2 (*p* < 0.001), but without statistically significant differences between genders. The lipid profile showed scarce differences in obese subjects, and non-significant variations were established in this state. The leptin values are also described in the same table. In this case, we observe a clear difference between genders and obesity. In both situations, the differences are markedly significant (obesity *p* < 0.005 and gender *p* = 0.062).

Table 3 shows the results of the Spearman’s correlation analyses regarding ANGPTL8 and the anthropometric and clinical variables in those patients included in this work. Results indicate that ANGPTL8 concentration was positively correlated with HDL-C levels (ρ  =  0.889, *p*  < 0.05) and HOMA-S (ρ  =  0.785, *p* < 0.05) in obese men. 

In the same way, ANGPTL8 was inversely correlated with insulin concentration (ρ  =  −0.785, *p* < 0.05) and HOMA-IR (ρ  =  −0.785, *p* < 0.05). In contrast, ANGPTL8 concentration was not correlated with the rest of clinical variables in all participants.

### 3.2. Variations in Plasma Levels of ANGPTL8 in Non-Obese and Obese Patients 

**Figure 1:**
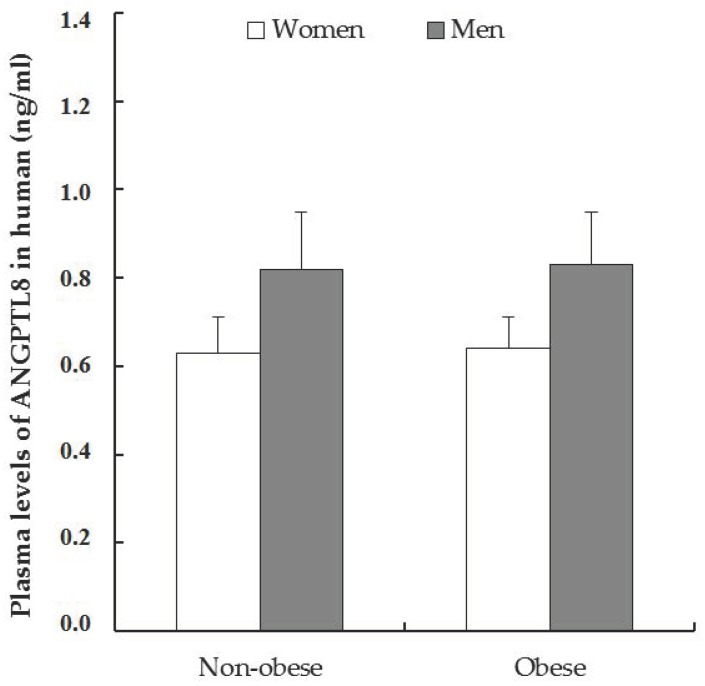
Plasma circulating levels of C-terminal 139–198 form of ANGPTL8. Data was expressed as mean ± the standard error of the mean (SEM), and the values in the non-obese women were 0.63 ± 0.10 ng/mL, *n* = 11 and obese women 0.64 ± 0.04 ng/mL, *n* = 33. In non-obese men 0.82 ± 0.09, *n* = 10 versus 0.83 ± 0.10 ng/mL in obese men, *n* = 14. The Student’s *t*-test analysis in non-obese patients (women vs. men) was *p* = 0.0763, and in obese patients (women vs. men) *p* = 0.176 (n.s.).

In this work, we determined the circulating concentration of the C-terminal 139–198 form of human ANGPTL8 in the plasma of patients by EIA. As is shown in Figure 1, ANGPTL8 levels in plasma were similar in non-obese and obese patients, but a weak increment was observed in men compared to women. Student’s *t*-test and one-way ANOVA did not show statistically significant differences among the groups.

### 3.3. Detection of ANGPTL8 in Human Adipose Tissue

**Figure 2:**
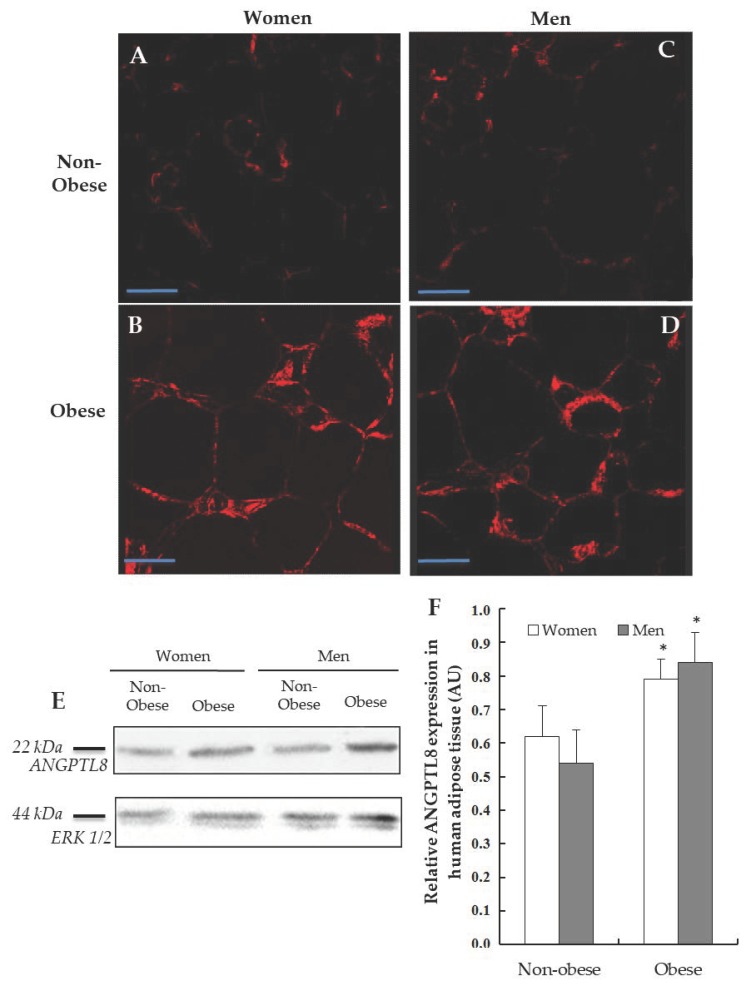
Detection of ANGPTL8 in human visceral adipose tissue (VAT). Representative images from the immunofluorescence reaction for positive ANGPTL8 (in red) in adipose cells of patients, (**A**, non-obese women, *n* = 4), (**B**, obese women, *n* = 4), (**C**, non-obese men, *n* = 4), (**D**, obese men, *n* = 4). Scale bar: 60 μm. (**E**) Western blotting analysing the expression of ANGPTL8 in human VAT (*n* = 6), ERK1/2 was used to confirm equal loading of samples. (**F**) Plot showing the relative ANGPTL8 expression as arbitrary units (AUs).

The area of the lipid vacuoles was measured as shown in Table 1. Obese patients presented a greater lipid drop, (women 4458.1 ± 80.2 μm^2^ and men 4376.1 ± 100.9 μm^2^), comparing with non-obese subjects (3958.6 ± 85.2 μm^2^ and 3984.9 ± 93.0 μm^2^, respectively), being statistically significant in obesity (*p* < 0.005). This data was corroborated in Figure 2 and this verified the classification made according to the nutritional status and the abdominal circumference. 

The next goal was the detection of ANGPTL8 protein in the human VAT by Western blotting (Figure 2E). The densitometric study of the bands obtained by Western blotting was carried out considering the expression of the protein ERK1/2 and the band area; then, the immunoreaction of ANGPTL8 was expressed in arbitrary units (AUs). Morbid-obese patients showed such ANGPTL8 protein expression higher than non-obese. When the results were analysed, significant differences were found (*p* < 0.05, Figure 2F). On the other hand, the presence of ANGPTL8 in histological sections of human adipose tissue using immunohistochemistry has been analysed. The micrographs A, B, C and D (Figure 2) show the immunoreactivity for ANGPTL8 in human adipocytes, separated by gender and nutritional state. 

The presence of ANGPTL8 is indicated by a red stain. As can be seen, ANGPTL8-immunoreactivity was localised in the cytoplasm, but its localisation in the surface of lipid droplets could not be ruled out. The protein was distributed throughout the cytoplasm in all of the groups studied, although there is a significant greater signal in obese patients (Figure 2B,D).

**Figure 3:**
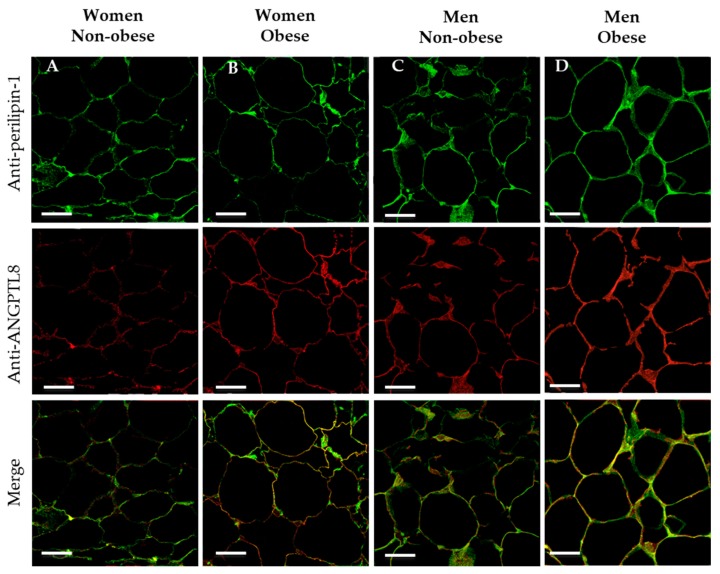
Co-existence of perilipin-1 and ANGPTL8 in human VAT. Representative images showing the double immunofluorescence reaction for positive anti-perilipin-1 (in green), ANGPTL8 (in red) and the merge in adipose cells of patients (in yellow), (**A**, non-obese women, *n* = 4), (**B**, obese women, *n* = 4), (**C**, non-obese men, *n* = 4), (**D**, obese men, *n* = 4). Scale bar: 63 μm.

On this matter, to determinate whether ANGPTL8 was colocalised on the surface of fat cells, double immunocytochemical labelling for ANGPTL8 and perilipin-1 (protein that coats lipid droplets in adipocytes) was carried out. In Figure 3 the presence of perilipin-1 is indicated by the green stain, and ANGPTL8 in red. The micrographs that present the merge of images show the co-existence of both proteins on the surface of adipocytes (in yellow colour). 

### 3.4. Gene Expression and Localisation of ANGPTL8 in Visceral Adipose Tissue of Non-Obese and of Obese Patients

**Figure 4:**
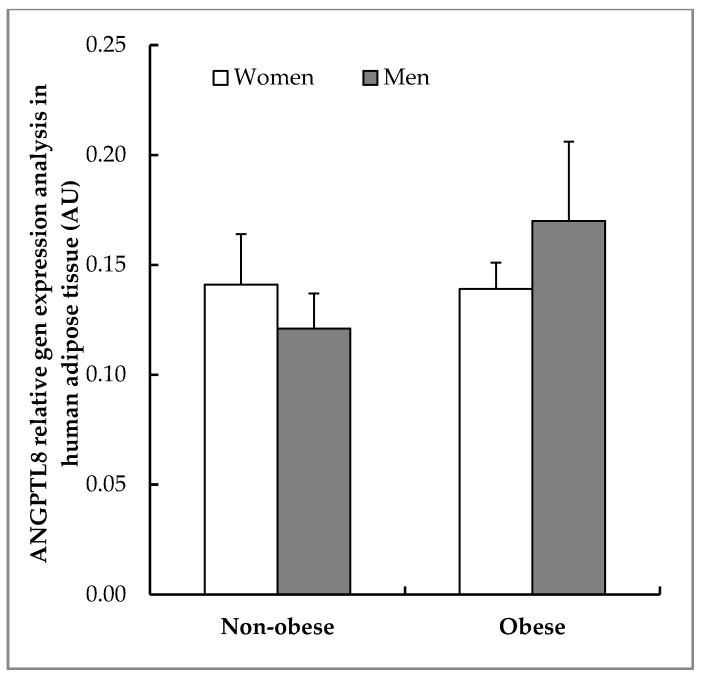
Expression of ANGPTL8 in human VAT. Real time polymerase chain reaction (RT-PCR) analysing ANGPTL8 relative gene expression in human VAT (non-obese women, *n* = 6; non-obese men, *n* = 5; obese women, *n* = 6; obese men, *n* = 5) calculated by normalisation to Ribosomal Protein L19 (RPL19) and expressed as arbitrary units (AUs).

In order to evaluate the synthesis of ANGPTL8 in VAT, RT-PCR for ANGPTL8 mRNA was carried out from the VAT of the same patients. As can be seen in Figure 4, ANGPTL8 mRNA was present in all of the conditions studied. Obese patients, mainly in men, have a slightly higher ANGPTL8 gene expression, but the differences observed in mean values were not statistically significant (*p* = 0.89). An important aspect of this expression is determinate where ANGPTL8 mRNA is located into the visceral adipose tissue. For answering this question, in-situ hybridisation was developed in samples of VAT from the same patients.

**Figure 5:**
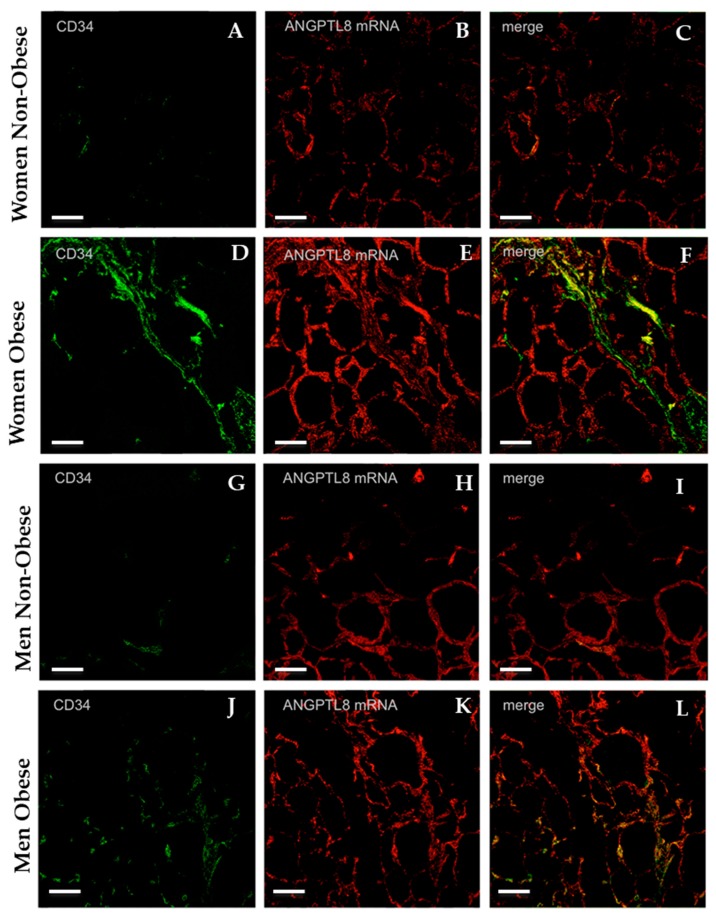
Representative images showing the immunofluorescence reaction for the endothelial cells’ marker anti-CD34 in adipose tissue (micrographs **A**, **D**, **G**, **J**, in green). The micrographs **B**, **E**, **H**, **K** present the in situ hybridisation for ANGPTL8 in VAT (ANGPTL8 mRNA, in red). The micrographs **C**, **F**, **I**, and, **L** are the merge of both images, (non-obese women, *n* = 4), (obese women, *n* = 4), (non-obese men, *n* = 4), (obese men, *n* = 4). Scale bar: 60 μm.

Figure 5 shows the results obtained from a combined study by immunocytochemistry for endothelial cells by using CD34 protein as marker for vascular endothelium (green colour), and in-situ hybridisation for ANGPTL8 mRNA (red colour). Endothelial cells were more abundant in the VAT of obese patients (Figure 5D,J) than in non-obese patients (Figure 5A,G). In-situ hybridisation (Figure 5B,E,H,K) labelling of adipocytes was evident in all groups analysed with higher labelling intensity in obese than in non-obese patients.

As can be observed in Figure 5C,F,I,L, coexistence for CD34 and ANGPTL8 mRNA was present, but it was more evident in obese than in non-obese patients (yellow colour). 

## 4. Discussion

It has been demonstrated that angptl8 is largely expressed in liver, and even in WAT, brown adipose tissue (BAT) and the adrenal glands in mice. In humans, it was only initially identified in the liver, although research has also brought to light its expression in the adipose tissue [13,39,40]. The ANGPTL family of proteins has been proposed to have a dual role in regulating triglyceride and glucose metabolism [33,35,36,41]. It has been well established that ANGPTL8 is a circulating factor secreted from the liver. However, it is unknown whether ANGPTL8 is also secreted from adipose tissue, although in WAT and BAT it is speculated that ANGPTL8 could function in a non-endocrine manner [35]. On this matter, exciting but conflicting data has emerged regarding the involvement of ANGPTL8, and as a result, ANGPTL8 has been suggested as a potential therapeutic target for dyslipidaemia and diabetes [6,42].

Many research groups have studied the plasma levels of ANGPTL8, finding important discrepancies in the values obtained, since two different types of kits were used to carry out the enzyme immunoassay. The first is aimed at the localisation of the N-terminal end, ignoring the presence of the fragments resulting from the protein degradation. The other type of kit is directed to the detection of the C-terminal end, locating not only the total length of the protein, but also the fragments that are produced after its degradation [43]. A meta-analysis conducted in 2016 analysed nine of these articles, reaching the conclusion that the differences observed among the kits could be mainly related to the characteristics of the analysed samples, since other individual factors studied can influence over the antibodies used for the analysis of ANGPTL8. Thus, it is possible that there are no direct differences between the two types of EIA kits used by the different research groups [44]. 

We have considered that the C-terminal fragments resulting from protein degradation could play some important biological functions, as suggested by Fu et al, 2014 [45]. For this reason, we have studied the circulating level of the C-terminal 139–198 forms of ANGPTL8. Our data shows that in subjects with morbid obesity and insulin resistance, there are not significant changes in the plasma levels of ANGPTL8 in relation to non-obese patients. These results are in concordance with that described in 2014 by Fenz et al., in morbid-obesity and insulin resistance individuals in which ANGPTL8/ betatrophin is unaltered, but it correlates significantly to atherogenic lipid profiles in high-risk cohorts [23]. However, other authors have reported conflicting results regarding the plasma levels of ANGPTL8 in overweight and obesity patients, since some had higher levels [45,46,47], and others lower levels [24,48,49], as shown in the meta-analysis conducted by Ly et al [44].

Besides, in our study the men presented a higher plasma concentration of this protein than women, but without statistically significant differences. This gender variation in ANGPTL8 levels is not ruled out, since data exists supporting it. In the Chinese population, non-obese and diabetic men present a higher concentration of this protein than women [50], and also children or adolescent males [51]. This could indicate that the plasma levels of ANGPTL8 would be influenced by the hormonal environment, like other adipokines, such as leptin or adiponectin, which present a sexual dimorphism, even though, in the concrete case of the ANGPTL8 protein, with the current data, it is not totally clarified [52].

On the other hand, we have studied the correlation of ANGPTL8 with some metabolic parameters; in our case we have found a positive correlation in obese patients of ANGPTL8 and HDL-cholesterol. This data is in agreement with other publications in which the change in ANGPTL8/betatrophin levels was positively correlated with the change in HDL-C concentrations in T2DM [18] and obese patients [53]. In obese women, it proposes that ANGPTL8 has a potential role in dyslipidaemia, and is intimately related to HDL [34]. 

This positive correlation could be another tool in dyslipidaemia treatment. However, other authors have not found a correlation between the lipid profile and ANGPTL8 [54], and even negative correlation [55]. 

In turn, obese subjects presented an increased adipocyte size, hypertrophied visceral adipose tissue and an elevated HOMA-RI index. We have observed a negative correlation between the ANGPTL8, insulin and homeostasis model assessment of insulin resistance (HOMA-IR). Studies in obese children or adolescents [56], obese adults, women with polycystic ovary syndrome (PCOS) and type 2 diabetes mellitus (T2DM), support our results, since ANGPTL8 were strongly and negatively correlated with all markers of insulin resistance [24,56,57]. These results suggested that metabolic status is an important regulator of circulating ANGPTL8/betatrophin levels. However, many clinical data have analysed the correlation of ANGPTL8 and the metabolic features of the patients, showing contradictory results. A positive correlation was found in overweight individuals, but not in individuals with obesity or T2DM [58,59]. For other authors, no significant relationships were observed among serum ANGPTL8/betatrophin levels and indices of insulin resistance or beta-cell function [18,60]. The disparity in the results could be due to the lack of homogeneity among the population included in the studies. Currently, it is speculated that the relationship among these parameters could be associated with indicators of cellular inflammation in these patients [61]. 

Nowadays it is unknown if the ANGPTL8 circulating plasma levels have the liver as the main source or, if the adipose tissue contributes to increase them. This latter speculation would be supported on the fact that in the obese patients, the ANGPTL8 plasma levels are higher than in non-obese patients. However, other important data suggest that circulating ANGPTL3 levels are much higher than ANGPTL8 levels. Both ANGPTL3 and ANGPTL8 are expressed in the liver, and thus, both could be secreted from the liver as a complex, enter into the circulation, and inhibit vascular LPL. In adipose tissue ANGPTL8 is also expressed, but it lacks ANGPTL3 expression, so ANGPTL8 would not be secreted efficiently from the adipose tissue [62]. 

By Western blotting and immunocytochemistry, our results demonstrate that ANGPTL8 was located in the adipose tissue in the cytoplasm and co-exist with perilipin-1, protein that coats lipid droplets in adipocytes. These results agree with other authors that have described a similar distribution along with cytoplasm, in the form of vesicles, with the highest density of these vesicles around the nucleus [63].

Besides, we have analysed ANGPTL8 mRNA expression and we have confirmed that this protein is expressed in human visceral adipose tissue. However, although some variations were found, we have not been able to establish significant differences between gender and nutritional state in the studied patients. In humans, ANGPTL8 sequence variations have been demonstrated to be associated with lipid profiles by Genome-Wide Association (GWA), and three ANGPTL8 single nucleotide polymorphisms (SNP) are strongly associated with lipid profiles [41]. 

The visceral adipose tissue obtained after laparoscopic surgery is not only composed of adipocytes, but also of connective and endothelial tissue. Therefore, the determination of gene expression by RT-PCR does not differentiate the place of synthesis. Accordingly, in-situ hybridisation is the best possible way to observe the structure where the specific mRNA is produced. Similar to what was found for the protein by immunocytochemistry, in-situ hybridisation showed that the mRNA that encodes ANGPTL8 was present in adipocytes, but furthermore, marking human endothelial cells with anti-CD34 have made clear to us the coexistence of ANGPTL8 mRNA in the endothelial cells of blood vessels of the adipose tissue. These results suggest that ANGPTL8 is synthesised and is located in the adipocytes of visceral adipose tissue without discarding its expression in endothelial cells.

In preliminary studies, other members of the ANGPTL family were observed in regions such as the aorta by in-situ hybridisation [64]. In primary cultures of preadipocytes, the non-adipocyte portion of the adipose tissue cell population (i.e., the stromal vascular fraction (SVF), which includes presumed preadipocytes) reveals that the ANGPTL8 transcript is exclusive to adipocytes with a lack of expression in stromal-vascular cells [9]. Up to now, there is no accurate evidence of the presence of ANGPTL8 protein in endothelial cells, nor of the mRNA that does encode it. 

The main function described for the ANGPTLs family is the inhibition of LPL, at the level of peripheral muscle cells, forming part of a more complex model that includes two other members of ANGPTL3 and 4. The balance between the activity in fasting and feeding states determines the correct metabolism of triacylglycerol, in both visceral adipose tissue and muscle. The Zhang model (2016) proposes that in fasting states, ANGPTL4 suppresses LPL in adipose tissue and prevents lipid storage, whereas after food intake, ANGPTL3 and 8 would act at the muscle to inhibit LPL and prevent lipid metabolism at this level. Simultaneously, in adipose tissue this enzyme is active for favouring energy storage [35]. At the same time, the authors explain limitations in this model, since one of the points to take into account is the null reference to the role played by the expression of ANGPTL8 in the visceral adipose tissue. 

LPL is synthesised and secreted by a limited number of cells that include cardiomyocytes and adipocytes, and upon release by these cells it is transported to the luminal side of the capillary endothelium by the GPIHBP1 (proteinglycosylphosphatidylinositol-anchored high density lipoprotein binding protein 1), where LPL remains anchored to the capillary wall [65]. Considering this fact, the binding of ANGPTL3 and ANGPTL8 to LPL-GPIHBP1 complexes on the surface of the vascular cells has been analysed, and it was suggested that ANGPTL8 has a role in LPL functional inhibition, but only when it acts jointly with ANGPTL3 [66,67]. 

Moreover angiopoietin-like proteins, specially ANGPTL3, are vascular growth factors that are highly specific for endothelial cells, and these perform a variety of other regulatory activities to influence inflammation, and have been shown to possess both pro-atherosclerotic and athero-protective effects [68]. However, so far there was no evidence that determined the expression of ANGPTL8 in the vascular tissue. Our study corroborates the presence of ANGPTL8 in adipocytes, and also presents clear evidence of its expression in the endothelial cells of human VAT.

On the other hand, it is not known if there is any mechanism by which ANGPTL8 is capable of crossing the endothelial cells, in order to reach the bloodstream. Janssen et al. [6] and Vatner et al. [42] suggest the importance of establishing the location in which the ANGPTLs proteins act. Its location, as explained above, is clearly necessary to know in-depth about how these proteins participate in lipid metabolism and the tolerance of glucose. At the same time, the knowledge of the site of action can help to the continuous studying of some functions of ANGPTL8 currently unknown. 

## 5. Conclusions

In summary, our data suggests that ANGPTL8 plasmatic levels do not change significantly in patients with morbid obesity, although there is a modest difference related to gender that we cannot explain with the current studies. Besides, the present study demonstrates that in visceral adipose tissue, the ANGPTL8 protein is well defined in the cytoplasm and surface of adipocytes, and we demonstrate that ANGPTL8 mRNA is present in the adipocytes and endothelial cells of obese and non-obese human VAT. These findings suggest the possibility that among other functions, ANGPTL8 could perform an either paracrine and/or endocrine role in the adipose tissue. 

In addition, although morbid obesity seems to be accompanied by an increase in the presence of ANGPTL8 in the adipose visceral tissue, this morbid obesity does not appear to be a relevant factor involved in the regulation of changes in mRNA for the ANGPTL8 synthesis in these cells. 

## Figures and Tables

**Table 1:** Anthropometric features of the non-obese and obese patients included in this study.

	Women		Men				
	Non-obese	Obese	Non-obese	Obese	P 2-*way*-ANOVA		
Patients	*n* = 22	*n* = 44	*n* = 20	*n* = 23	Obesity	Gender	Interaction
Age (years)	58 ± 14	48 ± 2	58 ± 8	47 ± 3	0.462	0.957	0.945
BMI (kg/m^2^)	21.7 ± 1.9	45.6 ± 1.3	27.3 ± 1.07	47.2 ± 3.0	<0.001	0.092	0.346
Waist circumference (cm)	88.8 ± 9.1	131.4 ± 2.7	77.5 ± 29.5	137.0 ± 6.2	<0.001	0.800	0.453
Lipid drop area (µm^2^)	3958.6 ± 85.2	4458.1 ± 80.2	3984.9 ± 93	4376.1 ± 100.9	<0.005	0.805	0.635

**Table 2:** Clinical and biochemical features of the non-obese and obese patients included in this study.

	Women		Men				
	Non-obese	Obese	Non-obese	Obese	P 2-*way*-ANOVA		
Patients	*n* = 22	*n* = 44	*n* = 20	*n* = 23	Obesity	Gender	Inter- Action
Glucose (mg/dL)	94.0 ± 8.7	101.6 ± 2.1	97.8 ± 4.7	98.3 ± 8.7	**0.105**	0.660	0.836
Insulin (μU/mL)	8.6 ± 1.4	18.2 ± 0.7	5.9 ± 1.7	24.7 ± 5.6	<0.001	0.456	0.063
HbA1C (%)	5.2 ± 0.1	10.6 ± 2.9	5.8 ± 0.7	11.6 ± 4.7	0.053	0.549	0.418
C-Peptide (ng/mL)	2.0± 0.4	4.3 ± 0.2	2.2 ± 0.4	5.8 ± 0.8	<0.001	0.286	0.459
HOMA β (%)	94.2 ± 16.1	134.2 ± 9.1	69.3 ± 10.2	175.4 ± 20.7	<0.001	0.590	0.031
HOMA-S (%)	88.7 ±12.3	41.8 ± 5.3	125.5 ± 11.6	32.4 ± 7.7	<0.001	0.133	0.012
HOMA-IR (AU)	1.1 ± 0.3	2.4 ± 0.2	0.8 ± 0.1	3.1 ± 0.6	<0.001	0.578	0.166
Triglycerides (mg/dL)	77.6 ± 16.8	129.0 ± 34.4	108.8 ± 8.4	163 ± 17.5	0.212	0.449	0.966
Cholesterol (mg/dL)	123.0 ± 13.6	154.7 ± 6.7	162.5 ± 31.0	158.4 ± 11.3	0.601	0.311	0.442
LDL-C (mg/dL)	63.1 ± 8.3	86.0 ± 4.4	94.3 ± 19.6	95 ± 7.6	0.526	0.526	0.54
HDL-C (mg/dL)	44.4 ± 12.8	42.8 ± 2.2	55.7 ± 12.2	34.6 ± 3.7	0.092	0.816	0.147
Leptin (ng/mL)	19.9 ± 8	71.7 ± 7.1	6.8 ± 0.2	28.1 ± 6.8	<0.005	0.062	0.134

**Table 3:** Spearman’s correlation analyses regarding ANGPTL8 and anthropometric and clinical variables in non-obese and obese patients.

ANGPTL8								
	Women				Men			
	Non-obese *n* = 22		Obese *n* = 44		Non-obese *n* = 20		Obese *n* = 23	
Variable	ρ	*p*	ρ	*p*	ρ	*p*	ρ	*p*
Age (years)	−0.3714	n.s	−0.2814	n.s	0.5429	n.s	−0,4762	n.s
BMI (kg/m^2^)	0.2000	n.s.	0.0625	n.s.	−0.8000	n.s.	0.5429	n.s.
Waist circumference (cm)	−0.3714	n.s.	−0.0760	n.s.	−0.8000	n.s.	0.1429	n.s.
Lipid drop area (µm^2^)	0.2723	n.s.	0.1345	n.s.	0.1429	n.s.	0.0952	n.s.
Glucose (mg/dL)	0.5429	n.s.	−0.2731	n.s.	0.0857	n.s.	0.1778	n.s.
Insulin (μU/mL)	0.5429	n.s.	−0.1313	n.s.	−0.1429	n.s.	−0.7857	*p* < 0.05
HbA1C (%)	−0.5000	n.s.	0.2284	n.s.	−0.7000	n.s.	−0.1627	n.s.
C-Peptide (ng/mL)	−0.9000	n.s.	−0.0315	n.s.	0.2855	n.s.	0.2571	n.s.
HOMA β (%)	0.0000	n.s.	0.0284	n.s.	−0.1429	n.s.	0.3929	n.s.
HOMA-S (%)	−0.3000	n.s.	0.2021	n.s.	0.2000	n.s.	0.7857	*p* < 0.05
HOMA-IR (AU)	0.3000	n.s.	−0.2021	n.s.	−0.2000	n.s.	−0.7857	*p* < 0.05
Triglycerides (mg/dL)	0.2000	n.s.	−0.0802	n.s.	−0.4857	n.s.	−0.5357	n.s.
Cholesterol (mg/dL)	−0.0857	n.s.	0.0385	n.s.	−0.3000	n.s.	−0.1786	n.s.
LDL-C (mg/dL)	0.05798	n.s.	0.0946	n.s.	−0.8000	n.s.	−0.5946	n.s.
HDL-C (mg/dL)	0.3143	n.s.	−0.2888	n.s.	0.4000	n.s.	0.8895	*p* < 0.05
Leptin (ng/mL)	0.7714	n.s.	0.0865	n.s.	−0.1000	n.s.	0.5000	n.s.
